# Breast tuberculosis in Northeast Iran: review of 22 cases

**DOI:** 10.1186/1472-6874-14-72

**Published:** 2014-05-31

**Authors:** Behnaz Khodabakhshi, Fatemeh Mehravar

**Affiliations:** 1 Infectious Diseases Research Center, Golestan University of Medical Sciences, Shastkola Road, Gorgan, Iran; 2 School of Public Health, Epidemiology and Biostatistics Department, Tehran University of Medical Sciences, 5TH Floor, Poorsina St, Keshavarz Ave, Postal Box: 14155–6446, Tehran, Iran

**Keywords:** Breast tuberculosis, Mammary tuberculosis, Tuberculosis treatment, IRAN

## Abstract

**Background:**

Breast tuberculosis (breast TB) is an extremely rare disease, so case reviews are also rare.

**Methods:**

This study is a retrospective review of patients with breast TB who were treated between 2002 and 2012 at the Health Center of Gorgan City.

**Results:**

All 22 patients were females, their mean age was 32.4 years, and all were new cases. Patients presented with swelling of the breast (22%), lump (55%) and excretion from the involved breast (27%), and breast pain (55%). The highest rate of breast TB occurred in 2011 (27%). All patients received the DOTS regimen for a mean duration of 7.3 ± 0.7 months; in addition, segmental resection was performed on 11 patients (50%).

**Conclusions:**

The findings confirmed that breast TB in Iran should be considered as a differential diagnosis of breast masses. All patients in our study received the daily and ‘Directly Observed Treatment Short-course’ (DOTS) regimens. Anti-tubercular therapy for six months with or without minimal surgical intervention currently is the main treatment.

## Background

The World Health Organization (WHO) estimated that there were 8.7 million cases of tuberculosis (TB) in the world in 2011, a rate of 125 cases per 100,000 people. It also was estimated that there were 12 million prevalent tuberculosis cases [1]. Breast TB was first defined by Sir Astley Cooper in 1829 as a rare form of extrapulmonary TB [2]. Breast TB is a very rare disease [3-5] and constitutes only 0.025-1.04% of all breast diseases [6]. Its prevalence has been estimated to be 0.1% of breast lesions examined histologically, and it constitutes about 3–4.5% of surgically-treated breast diseases in developing countries [7]. Despite the high prevalence of tuberculosis, mammary cells offer great resistance to the survival and multiplication of mycobacterium tuberculosis [8]. The disease may be of primary etiology when infection affects only the breast, or it may result from other foci in the body, which is termed as secondary breast TB. Presentation of breast TB is variable and may be confused with other disorders [9,10]. Fine Needle Aspiration Cytology (FNAC) is the most extensively used initial invasive method for diagnosis of breast TB [11,12], and the ‘gold standard’ diagnostic tool for breast TB is bacteriological culture of breast tissue or the Ziehl–Neelsen stain [10]. Breast TB is often misdiagnosed as a pyogenic abscess or carcinoma of breast, both clinically as well as radiologically, especially if well-defined clinical features are absent [13]. In the present study, our aim was to evaluate demographic data, diagnostic methods, and therapeutic regimens in 22 breast TB patients treated at the Tuberculosis Control Health Center in Gorgan, Iran over a period of 10 years.

## Methods

Over the 10-year period from 2002 through 2011, 22 patients were diagnosed with breast TB, and the patients were referred to health centers in Gorgan City for the ‘Directly Observed Treatment Short-course’ (DOTS). Gorgan is in the center of Golestan state in northern Iran, just southeast of the Caspian Sea. Demographic data, such as age, gender, weight at diagnosis, ethnicity, location, occupation, marital status, educational status, the family’s and the patient’s TB history, complaints, and physical examination findings of the patient records were documented.

The cytological findings of epithelioid cell granulomas, Langhans’ giant cells, and lymphohistiocytic aggregates confirmed the diagnosis. The discharge from the sinus was subjected to Ziehl-Neelson staining and cultured for acid fast bacilli. Sonography was performed for 77.2% of the patients. Mammography was performed on three patients (13.6%), and non-specific stromal coarsening existed in all three patients. The chest radiographs of the 22 patients were normal. The diagnosis of breast TB was confirmed by a combination of clinical suspicion and biopsy findings. After primary diagnosis of patients by our doctor, they were referred to a health center in Gorgan and continued treatment as planned.

In 1978, according to the World Health Organization’s (WHO’s) guideline [1], the Ministry of Health and Medical Education of Iran effectively implemented the DOTS strategy to fight tuberculosis. Based on the national strategy of DOTS, new patients were treated with a short-term diet for six months, followed by re-treatment with a regimen of eight months. Both regimens consisted of two phases, i.e., attack and maintenance.

The DOTS regimen was put into practice in Gorgan in June 1998. The records of the breast TB cases that were treated between January 2002 and June 2012 were reviewed. The breast TB regimens used in the health centers in Gorgan City are presented in Table 1. In this study, standard six-month anti-TB therapy (isoniazid, rifampicin, pyrazinamide, and ethambutol) was commenced with good clinical response. The continuation phase lasted four months with isoniazid and rifampin on a daily basis and the regime’s maintenance phase continued for another three months.

**Table 1:** **Breast TB treatment regimens **[11]

**Treatment time**	**Initial phase regimens**	**Continuation phase regimens**
**6 months**	2 months of isoniazid, rifampycine, pyrazinamide, ethambutol or streptomycin	4 months of isoniazid and rifampycine
**9 months**	2 months of isoniazid, rifampycine, pyrazinamide, ethambutol or streptomycin	7 months of Isoniazid and rifampycine

Some patients had breast lumps that were potentially malignant, and they also underwent segmental mastectomy. Residual lumps after anti-tubercular therapy (ATT) may require surgical removal. Minimal surgical intervention was required for drainage of abscesses of the breast or taking biopsies from the wall of the abscess, scraping of sinuses in the breast, and incisional or excisional biopsy.

This study obtained ethics approval and was approved by Ethical Committee of Golestan University of Medical Science.

### Statistical analysis

Data relating to the duration of therapy and to whether DOTS was administered were recorded with SPSS version 16.0. Descriptive analyses were performed to detect differences between subgroups.

## Results

In this study, 22 known breast TB cases were identified, and all the patients were female. The mean age of the patients was 32.4 (range 23–66). All of the patients were new cases. Among the patients, 15 were of reproductive age, and one of the 15 was pregnant at the time of diagnosis.

Patients presented with swelling of the breast (22%), lump (55%) and excretion from the involved breast (27%), and breast pain (55%). The left side of the breast was involved in 16 cases, and the right side of the breast was involved in 4 cases. The disease was bilateral in two patients. Most of the patients (59.09%) were from urban areas, and most were of Persian ethnicity (68.18%), as summarized in Table 2. The highest rate of breast TB was seen in 2011 (27%).

**Table 2:** Demographic and clinical data of the breast tuberculosis (TB) patients (n = 22)

**Variables**	**N (%)**	
**Marital status**	Single	1 (4.54)
	Married	21 (95.4)
**Occupation**	Housekeeper	17 (77.27)
	Employee	2 (9.09)
	Farmer	2 (9.09)
	Animal husbandry	1 (4.54)
**Educational status**	Graduates	6 (27.27)
	Diploma	8 (36.36)
	Under Graduates	5 (22.72)
	bachelor	3 (13.63)
**Ethnicity**	Persian	15 (68.18)
	Sistani	3 (13.63)
	Turk	3 (13.63)
	Turkmen	1 (4.54)
**Multiplicity of delivery**	Zero	1 (4.54)
	1	7 (31.81)
	2	8 (36.36)
	3 and more	5 (22.72)
**Breast-feeding history of Involved in breast**	Had	16 (72.72)
	No	5 (22.72)
**Location**	Urban	13 (59.09)
	Rural	8 (36.36)
**History of contact with tuberculosis**	Had	5 (22.72)
	No	11 (50)
	Suspicious	6 (27.27)
**Treatment**	Anti-TB drugs	11 (50)
	Surgery and anti-TB drugs	11 (50)
**Method of diagnosis**	Excisional Biopsy	18 (81.81)
	Culture positivity	2 (9.09)
**Involved part of the breast**	Left	16 (72.72)
	Right	4 (18.18)
	Bilateral	2 (9.09)
**Signs in the breast**	Swelling	5 (22.72)
	Mass	13 (59.09)
	Pain	12 (54.54)
	Excretion from the involved breast	6 (27.27)

Sonography was conducted for 17 patients, and a defined breast lump was found in 13 patients (55%); defined isolateral auxiliary lymph node involvement was observed in seven patients (31.8%); defined non-specific findings were made in five patients (18.5%), including hypoechoic masses, internal echoes, and irregular borders; and bilateral auxiliary lymph node involvement was observed in one patient (3.7%). Mammography was performed on three patients (13.6%), and the results showed non-specific stromal coarsening in all three patients. The most frequently used diagnostic tool was excisional biopsy (81.81%) and positive TB culture (9.09%). The breast TB diagnostic criteria for this study were based on biopsy and clinical response. Histopathology of the specimen (lump) findings included epitheloid cell granulomas without caseous necrosis.

Eleven patients underwent segmental mastectomy and anti-tuberculosis drug therapy, and another eleven patients underwent just anti-tuberculosis drug therapy after fine-needle aspiration. All patients completed therapy with a full recovery. Our treatment success rate was 100%. A six-month treatment regimen was administered to all patients, but for some patients (n = 4) that had a fistula and abscess, the regime was nine months. Twenty-two patients received the DOTS regimen for a mean duration of 7.3 ± 0.7 months; in addition, segmental resection was performed on 11 patients (50%), and two patients underwent complementary surgery for drainage of abscesses.

## Discussion

Although tuberculosis is a very common disease in endemic areas, isolated involvement of the breast is very rare [12]. Breast tissue, along with the skeletal muscle, appears to be relatively resistant to tuberculosis infection. Incidence of breast TB accounts for less than 0.1% of all breast lesions in Western countries and 4% of all breast lesions in TB endemic countries [14].

Breast TB usually affects young, lactating, multiparous women, though it also may occur in prepubescent males and in elderly women [15]. The mean age of the patients in our series was 32.4 years, 15 of the women were of reproductive age, and one of the 15 was pregnant at the time of diagnosis. Twenty-one of the 22 patients were married.

According to one study [16], the rate of involvement of the right and left sides was very similar with a slight dominance of the left breast. In contrast, Da Silva and Pal [17] reported beat TB mostly in the right breast, but Khanna [18] reported that the occurrence was the same on both sides. In our study, 16 out of 22 patients had breast TB in the left breast.

The mean duration of the symptoms was reported to be a few weeks or months, and it is generally less than a year, and late presentation is generally a problem in patients with breast TB [4]. Khanna and colleagues [18] reported the presentation at 8.5 months, whereas Tiwari and colleagues reported 9.4 months [4]. It was 8.5 months in our present study. In our opinion, the duration of symptoms was longer than has been reported in the literature.

breast TB may be primary or secondary, and the three modes by which it spreads are hematogenous, lymphatic, or direct [19]. In one study [16], only 1 of 27 cases with breast TB had a history of contact with a TB patient, while the others had no history of contact with TB. In another study [20], 5 of 7 cases had a history of contact with a TB patient. In our study, five of the 22 women with breast TB had a history of contact with a TB patient, nine had no such history of contact, and six were uncertain. In our opinion, having a history of contact with a TB patient should be considered in screening or examining patients for breast TB.

In breast TB, the presenting signs and symptoms tend to be breast masses and an open wound accompanied by discharge [21]. In our study, breast masses were the most frequent finding upon examination. In the majority of patients, the initial complaint was pain in the breast.

Tests are useful in the diagnosis and further evaluation of patients with breast TB. The ‘gold standard’ for the diagnosis of breast TB is the detection of M. tuberculosis by Ziehl–Neelsen staining or by culture [22]. Radiological imaging modalities, such as mammography or ultrasonography, are unreliable in distinguishing breast TB from carcinoma because of the variable pattern of presentation of such an inflammatory lesion [18]. Culture or PCR is not very sensitive, and this may cause some additional delays for diagnosis and under-diagnosis [23]. In the present study, the most frequently-used diagnostic tool was excisional biopsy (81.81%) and positive TB culture (9.09%).

Therapeutic guidelines indicate that a six-month regimen of anti-TB therapy is sufficient [24]. Surgical intervention is indicated in cases that show poor response to anti-TB therapy, and it is used mainly to drain abscesses or the excision of residual lumps. Simple mastectomy is used for cases with extensive disease that causes a large, painful, ulcerated mass that involves the entire breast [19]. In our study, 22 patients received the DOTS regimen for a mean duration of 7.3 ± 0.7 months; in addition, segmental resection was performed in 11 patients (50%). Our treatment success rate was 100%.

## Conclusion

Due to the high level of tuberculosis in Golestan Province, breast TB should always be included in the differential diagnosis in cases of inflammatory breast lesions and breast tumors. The use of anti-tuberculosis drugs, in combination with surgical drainage, usually produces an excellent outcome.

## Competing interests

The authors declare that they have no competing interests.

## Authors’ contributions

BK and FM contributed to the conception and design of the study, the acquisition of data and to the analysis and interpretation of data. FM drafted the manuscript. Both authors were involved in critically revising the article for important intellectual content and gave final approval of the version to be published. Both authors read and approved the final manuscript.

## Pre-publication history

The pre-publication history for this paper can be accessed here:

http://www.biomedcentral.com/1472-6874/14/72/prepub
